# Combining approach bias modification with working memory training during inpatient alcohol withdrawal: an open-label pilot trial of feasibility and acceptability

**DOI:** 10.1186/s13011-019-0209-2

**Published:** 2019-06-06

**Authors:** Victoria Manning, Katherine Mroz, Joshua B. B. Garfield, Petra K. Staiger, Kate Hall, Dan I. Lubman, Antonio Verdejo-Garcia

**Affiliations:** 1 Monash Addiction Research Centre and Eastern Health Clinical School, Monash University, 110 Church Street, Richmond, Victoria 3121 Australia; 2 Turning Point, Eastern Health, 110 Church Street, Richmond, Victoria 3121 Australia; 3 School of Psychology, Deakin University, Locked bag, Geelong, 2200 Australia; 4 Centre for Drug use, Addictive, and Anti-social Behaviour Research (CEDAAR), Deakin University, Locked bag, Geelong, 2200 Australia; 5 School of Psychological Sciences & Monash Institute of Cognitive and Clinical Neurosciences, 18 Innovation Walk, Clayton Campus, Wellington Road, Monash University, Melbourne, Victoria 3800 Australia

**Keywords:** Alcohol use disorder, Working memory, Approach bias, Cognitive bias, Cognitive training, Detoxification, Withdrawal

## Abstract

**Background:**

According to contemporary neurocognitive models, addiction is maintained by the biasing of information-processing and decision-making systems towards relatively automatic, impulsive, reward-seeking responses to drug-related stimuli, and away from more controlled, deliberative, “reflective” states of processing that could result in decisions to delay or avoid drug use. Cognitive training programs aimed at either countering “impulsive” processing or enhancing “reflective” processing alone have shown promise. However, there has been no attempt to simultaneously target both aspects of processing with a combined training program. We aimed to test the feasibility and acceptability of a novel ‘dual-training’ program targeting both processes during residential alcohol withdrawal, and to measure abstinence rates following discharge.

**Methods:**

Thirty-seven patients undergoing alcohol withdrawal at a residential unit participated in this open-label pilot feasibility study. We tested a 4-session program of dual cognitive training targeting both impulsive (approach bias) and reflective (working memory) aspects of processing. Descriptive statistics were used to examine feasibility (training uptake and completion rates) and acceptability (withdrawal from the study; participants’ ratings of the tasks). Alcohol abstinence rates were examined 2-weeks post-discharge.

**Results:**

Seven participants withdrew after commencing training. Twenty-six (70%) completed the 4-session training protocol, and four completed 3 sessions before discharging. Among participants who provided ratings, nearly all (93%) rated the training as interesting. Most (87%) indicated that they felt it had improved their attention. However, most did not feel it had decreased their craving for alcohol. At 2-weeks post-discharge, 16 (53%) participants reported abstaining from alcohol. For comparison, an earlier pilot trial in the same setting found a 68% abstinence rate with approach bias training alone, and 47% abstinence in a non-training control group.

**Conclusions:**

Dual training during residential alcohol detoxification appears to be both acceptable and feasible, suggesting that future research is warranted to test its effectiveness at reducing likelihood of relapse.

## Background

Contemporary neurocognitive models suggest that in alcohol use disorder (AUD), information-processing and decision-making are biased towards relatively automatic, implicit processes which drive initial approach tendencies [1, 2]. According to these models, normative information processing involves a series of iterations which progressively allow more associations and contextual information to be activated, enabling evaluation and response selection to move from more automatic to more reflective styles. In AUD, however, rapidly-activated, automatic impulses become sensitised to alcohol-related stimuli, resulting in strong activation of alcohol-approach tendencies, (i.e. approach bias [3, 4]), before more reflective processing styles are engaged. The ability to engage reflective processing may be further undermined by the neurocognitive deficits that are common among people with AUD [5, 6]. Increased approach bias has been found to have cross-sectional associations with increased hazardous drinking [7, 8], and to prospectively predict increased drinking after treatment in problem drinkers seeking to reduce their drinking [9], although one study found the opposite effect (lower approach bias predicting relapse) [10]. Importantly, however, approach bias can be reduced in alcohol dependent individuals through computerised approach bias modification (ABM) training, which has been shown to lead to reduced relapse rates [11–14]. Indeed, a recent systematic review suggests that ABM is effective in reducing a range of addictive behaviours [15], including alcohol use when applied as an adjunctive approach for individuals with AUDs [16].

Impairment in working memory, which refers to the ability to maintain and manipulate goal-relevant information, and which is thus implicated in reflective control [17], is one of the neurocognitive deficits most commonly observed among people with AUD [5, 6]. Such WM deficits make it harder to implement strategies to circumvent habitual behaviours, such as impulsive alcohol-approach tendencies, and can therefore hamper treatment outcomes [18]. Research has shown poorer WM among individuals with higher alcohol approach bias [19]. Cognitive training designed to improve WM capacity has shown promising results, including improved performance on WM tasks [20], reductions in delay discounting (the tendency to prioritise immediate small rewards over larger, delayed rewards) [21], and reductions in heavy drinking, particularly among individuals with high approach bias [22]. Therefore, strategies that both dampen approach bias and strengthen WM could improve treatment outcomes, yet to date this has not been explored, despite the fact that both ABM and WM training are low-cost, non-invasive, easy-to-administer interventions.

Treatment for AUD often commences with inpatient detoxification. Research has shown that the initial weeks of abstinence are a period of neuroplasticity and neural reorganisation [23–25], making this a potentially opportune time to modify neurocognitive processes. However, if detoxification is not followed by post-withdrawal treatment, around 85% of patients relapse [26]. Early relapse prevents subsequent engagement in post-withdrawal treatment. This highlights the importance of examining adjunctive interventions during residential withdrawal to prevent rapid relapse and increase the chance of ongoing post-withdrawal treatment engagement.

Our pilot study of a 4-session course of ABM during detoxification significantly reduced rates of early relapse relative to a control condition [12]. We expected that adding WM training to ABM (“dual-training”) would increase abstinence rates even further. Nevertheless, before executing costly randomised controlled trials, it is important to establish whether dual-training is acceptable and feasible (e.g., tolerated and completed) during detoxification treatment. The aim of the current pilot study was to examine the feasibility and acceptability of a novel computerised ‘dual-training’ program combining ABM and WM training during inpatient withdrawal, and examine rates of abstinence at 2-week follow-up relative to those observed in our earlier study with ABM-alone or sham-training.

## Methods

### Design and setting

We conducted an open-label single-group feasibility study in a residential alcohol and other drug withdrawal unit in the Melbourne metropolitan area. We aimed to measure outcomes related to feasibility (rate of uptake, rates of training completion) and acceptability (rate of withdrawal of consent to participate, participants’ ratings of the training tasks). We also conducted a 2-week follow-up to assess alcohol use following discharge from withdrawal treatment.

### Participants

**Fig. 1:**
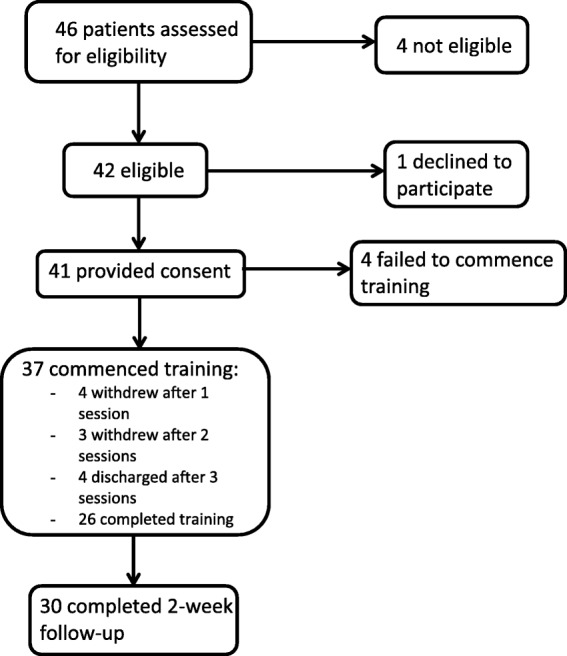
Recruitment and participant flow diagram

Inpatients undergoing alcohol withdrawal were assessed for eligibility in consultation with nursing staff. Those deemed eligible (*N* = 42) were approached on the fourth day of detoxification. Forty-one provided consent, of whom 37 commenced training (as shown in Fig. 1). Inclusion criteria were: alcohol as the primary drug of concern; aged 18–60 years; able to understand English; and weekly or more alcohol use in the past month. Exclusion criteria were: a diagnosed intellectual disorder; neurological illness; traumatic brain injury with loss of consciousness exceeding 30 minutes; or current psychotic episode (as assessed by clinical staff).

### Materials

#### Alcohol use

Participants’ alcohol use was measured using the Timeline Follow-back (TLFB) interview method [27]. At the baseline interview, participants were asked to recall their alcohol consumption in the 30 days prior to admission. At the 2-week follow-up, the TLFB was conducted via telephone interview, covering the 14 days following discharge from detoxification.

#### Acceptability

Acceptability of the dual-training program was informed by a three-item measure administered after completing training. Items were ‘I found the task improved my attention’, ‘I found the task decreased my craving for alcohol’ and ‘I found the task interesting’, with response options of ‘Strongly Agree’, ‘Agree’, ‘Unsure’, ‘Disagree’, and ‘Strongly Disagree’.

#### Alcohol craving

The Alcohol Craving Questionnaire – Short Form – Revised (ACQ-SF-R) [28] was administered before participants commenced the first session of training and after their final session of training. Scores on the ACQ-SF-R can range from 1 to 7, with higher scores indicating stronger cravings.

#### Severity of alcohol dependence

The Severity of Alcohol Dependence Questionnaire community sample version (SADQ-C) [29] was used to measure symptoms of physical dependence on alcohol. Scores on the SADQ-C can range from 0 to 60, with higher scores indicating greater severity of dependence. A score of 16–30 is believed to indicate moderate dependence and a score over 30 indicates severe dependence.

### Interventions

#### Approach Bias Modification Training (ABM)

We used a training version of the Alcohol Approach-Avoidance Task [14] implemented with E-Prime 2.0 software. Participants were instructed to pull a joystick in response to pictures in a horizontal (landscape) orientation, and push a joystick in response to pictures in a vertical (portrait) orientation. Pulling the joystick caused the image to expand on the screen, making the image seem to move “towards” the participant. Pushing the joystick caused the image to shrink, making it appear to recede into the distance. Images of 40 alcoholic and 40 non-alcoholic beverages commonly available in Australia were used, with each image appearing 3 times per session (i.e. 240 trials per session). Landscape-oriented pictures always contained images of alcoholic beverages. Portrait-oriented images always contained pictures of non-alcoholic beverages. Each session lasted approximately 15 min.

#### Working memory training

Participants completed five WM tasks each day, administered through the training application ‘Brainwell’, a program created by Monclarity LLC [30]. Prior to commencing the first training session, participants completed a cognitive ‘fitness test’ involving three 60-s tests of memory, attention, and problem solving, to calibrate initial difficulty to their level of functioning. The training tasks were gamified and adaptive, increasing and decreasing in difficulty in real-time depending on performance. Similar to facets of WM targeted by other training studies, [21, 22, 31] the tasks aimed to strengthen monitoring and updating abilities, spatial span, memory capacity, and visuo-spatial WM. The 10-trial spatial span task and the 5-trial monitoring and updating task each took approximately 5 min. The remaining 3 tasks (one memory capacity task and two visuo-spatial WM) each required participants to complete as many trials as possible in a 3-min period.

### Procedure

A researcher conducted sessions in a private room inside the detoxification unit. Baseline assessment and the first training session occurred on day four of detoxification. Participants completed a demographic information form, followed by the TLFB. Participants then commenced. Daily training sessions continued on the following 3 days, with the fourth and final session occurring on day 7 of detoxification. Each session involved WM training followed by ABM. The fourth session of training was followed by the acceptability questionnaire. Acceptability was also indexed by the number of participants who withdrew consent to participate after commencing training. Feasibility of the intervention was indexed by the rate of uptake and the rate of completion of the 4-session protocol. Two weeks following discharge, alcohol use was assessed in a telephone interview.

### Statistical analyses

Descriptive statistics were generated using IBM SPSS version 25.

## Results

Participants’ baseline characteristics are shown in Table 1.

**Table 1:** Participants Characteristics

	*M/n*	*SD/*%
Age (M years /SD)	42.7	8.9
Gender (*n*/% female)	17	46
Born in Australia (*n*/%)	32	89
Any post-secondary education (*n*/%)	22	59
Receiving unemployment or disability support benefits (*n*/%)	17	46
Relationship status (*n*/% single)	21	57
Any psychiatric comorbidity^b^ (*n*/%)	32	87
Family history of AUD (*n*/%)	24	65
Total drinking days in past month (M/SD)	27.2	5.4
Standard drinks^a^ in the past month (M/SD)	497.0	263.3
SADQ-C score (M/SD)	28.6	12.5
Anti-craving medications at admission (*n/%*)^c^	15	44

*AUD* alcohol use disorder, *SADQ-C* Severity of Alcohol Dependence Questionnaire (community version), *SD* standard deviation

^a^In Australia, a ‘standard drink’ is defined as 10 g (i.e. 12.7 ml) of pure alcohol

^b^Psychiatric diagnoses included depression (*n* = 24), anxiety disorders (*n* = 24), bipolar disorder (*n* = 7), post-traumatic stress disorder (*n* = 3), and other disorders (*n* = 3)

^c^Data on whether anti-craving medications were administered at admission was missing for 3 participants

### Feasibility

Of 42 patients invited to participate, only 1 patient declined participation and 37 of the 41 (90%) who agreed to participate commenced training. Of these 37 participants, 26 (70%) completed all 4 sessions of training. Four participants completed only 1 session, three completed 2 sessions, and four completed 3 sessions. These rates of uptake and completion suggest good feasibility of delivering dual training during detoxification.

### Acceptability

Seven (18%) of the 37 participants who commenced training withdrew consent prior to completing training. Task ratings from the 30 participants who did not withdraw, shown in Table 2, indicated that a large majority found the tasks interesting (93% either ‘strongly agreed’ or ‘agreed’) and felt they improved their attention (87% either ‘strongly agreed’ or ‘agreed’). This suggests that training was acceptable to a large majority of participants. However, the majority (53%) were ‘unsure’ if the training reduced their alcohol craving. Nevertheless, ACQ-SF-R scores revealed that there was a small but significant reduction in alcohol craving between the first (mean = 3.7) and final sessions (mean = 3.0) of training (t (29) = 3.788, *p* < .001).

**Table 2:** Percentage of participants endorsing each rating of the training tasks (*N* = 30)

	Strongly agree	Agree	Unsure	Disagree	Strongly Disagree
I found the task interesting	50	43	3	0	3
I found the task improved my attention	23	63	13	0	0
I found the task decreased my craving for alcohol	7	20	53	20	0

### Alcohol abstinence

Of the 30 participants who completed the 2-week follow-up, 16 (53%; 95% CI: 36–70%) reported abstaining from alcohol in the 14-days following discharge. Nonetheless, this was only slightly (6%) higher than we observed with sham-training in our previous pilot of ABM alone [12].

## Discussion

The study findings suggest that engaging patients in computerised WM and ABM training during residential alcohol withdrawal treatment is feasible. Most participants found dual training to be acceptable, rating it as interesting and helpful in terms of perceived improved attention. The two-week abstinence rate was similar to what we previously observed following no (‘sham’) training in our earlier study [12] and lower than we observed with ABM alone (absent WM training). However, it is important to note that this was a feasibility study which was not powered to examine effectiveness, and the estimated abstinence rate may lack precision due to the small sample size (note the 95% confidence interval extends from 36 to 70%).

We expected that adding WM training to ABM (dual training) could enhance abstinence rates, given that previous studies supported the effectiveness of WM training for substance use disorders [22, 32]. However, these previous studies adopted multi-session training protocols (e.g., 25 sessions delivered over one month). Thus, our 4 sessions were likely insufficient to have a positive impact on relapse rates, and future studies should consider testing this approach in settings where longer training is possible (e.g. post-withdrawal rehabilitation), or use post-discharge “booster” training sessions. Our findings regarding feasibility and acceptability suggest that a large proportion of patients would engage with such neurocognitive interventions, making them a worthwhile investment for future research. Limitations of this study include the lack of acceptability questionnaire assessments among the 7 participants who withdrew (meaning acceptability may be over-estimated). Moreover, the acceptability questionnaire was just a 3-item instrument that provides very limited feedback, and did not distinguish between the ABM and WM training. Self-reported alcohol outcomes were also not objectively-verified.

## Conclusions

Our findings indicate that dual-training is acceptable and feasible during alcohol withdrawal. We cannot draw conclusions regarding the effectiveness of this intervention from a small single-group, open-label study. However, considering the likelihood that WM training requires more than 4 sessions to be effective, we would suggest that larger trials designed to test the efficacy of this dual-training approach either be in treatment settings where longer courses of WM training are possible (e.g., post-withdrawal rehabilitation programs), or incorporate post-discharge “booster” sessions into their protocol.

## Abbreviations

ABM: Approach bias modification
AUD: Alcohol use disorder
TLFB: Timeline follow-back
WM: Working memory
